# The Evolving Role of Radiofrequency Guided Localisation in Breast Surgery: A Systematic Review

**DOI:** 10.3390/cancers13194996

**Published:** 2021-10-05

**Authors:** Salim Tayeh, Umar Wazir, Kefah Mokbel

**Affiliations:** London Breast Institute, Princess Grace Hospital, Hospital Corporation of America (HCA), 42-52 Nottingham Place, London W1U 5NY, UK; salimtayeh@hotmail.co.uk (S.T.); umarkhanwazir@gmail.com (U.W.)

**Keywords:** breast cancer, localisation, occult, radio-frequency tags, non-palpable breast lesions

## Abstract

**Simple Summary:**

A large portion of breast lesions are not palpable and need to be marked before surgery in order to aid complete surgical removal. Currently, this is accomplished by placing a hook in the lesion with radiological guidance, which is in turn attached to a wire through the skin. This is a well-understood technique and is called wire-guided localisation (WGL). It has drawbacks, including being uncomfortable for the patient, and the need for the wire to be placed within less than a day of the surgery. This is why certain wireless techniques have been developed to replace WGL. LOCalizer™ is one such technique, which uses radio-frequency identification tags. In this study, we have systematically reviewed the literature regarding LOCalizer™, and confirmed that it is a valid alternative to WGL. We have also highlighted its limitations and suggested potential technical refinements to improve its clinical performance.

**Abstract:**

Wire-guided localisation (WGL) has been the gold-standard for localising non-palpable breast lesions before excision. Due to its drawbacks, various wireless alternatives have been developed, including LOCalizer™, which is based on radio-frequency identification (RFID) technology. In this systematic review, we consulted EMBASE, Medline and PubMed databases using appropriate search terms regarding the use of RFID technology in the localisation of occult breast lesions. Retrospective and prospective studies were included if they quoted the number of patients, rate of successful placement, retrieval rate, margin positivity rate and the re-excision rate. In addition, studies comparing RFID to WGL were also included and analysed separately. Seven studies were included in this systematic review spanning 1151 patients and 1344 tags. The pooled deployment rate was 99.1% and retrieval rate was 100%. Re-excision rate was 13.9%. One complication was identified. Two studies compared RFID with WGL (128 vs. 282 patients respectively). For both techniques the re-excision rate was 15.6% (20/128 vs. 44/282 respectively, *p* value is 0.995). Based on our review, LOCalizer™ is safe and non-inferior to WGL in terms of successful localisation and re-excision rates. However, further research is required to assess the cost effectiveness of this approach and its impact on the aesthetic outcome compared with WGL and other wire free technologies to better inform decision making in service planning and provision.

## 1. Introduction

Non-palpable lesions form a large plurality of breast cancer cases treated every year. Recognition of the peculiar diagnostic challenge posed provided impetus to the development of mammography in the 1950s, which has made breast cancer screening possible [1,2]. Breast screening does lead to detection of non-palpable lesions at an earlier, more treatable stage, which would require preoperative localisation of the detected lesions [3].

The current gold standard, which has been prevalent since the 1970s, is the placement of hooks at the site of the lesion marked by wires protruding from the skin. This is commonly termed as wire-guided localisation (WGL) of the occult breast lesion [4,5,6].

The professional community at large has significant experience and familiarity with this technique, which is not to say that WGL is not without its drawbacks. It is uncomfortable for the patient. It carries a risk of needle-stick injury for both the surgeon and the radiologist. Dislodgement, fracturing and migration of the wire are all well-known possible issues which could arise. However, the most salient reason to find an alternative to WGL is the onerous scheduling requirement imposed on health systems by closely coupling radiological appointments with surgery slots [4,7,8].

Several wireless localisation techniques have been developed recently for the localisation of occult breast lesions. While they are still awaiting wider acceptance, their potential benefits in terms of patient satisfaction and reduction of strain on hospital scheduling systems could not be ignored.

We have recently investigated the clinical performance of currently available radiation-free wireless localisation technologies, specifically SAVI SCOUT (Merit Medical, Alieso Viejo, CA, USA) [9] and Magseed (Endomag Limited, Cambridge, UK) [10]. We have also systematically reviewed the literature regarding the currently available wireless localisation systems. SAVI SCOUT is based on reflectors using radar technology [11]. Magseed utilises ferromagnetic markers which are located by handheld proprietary magnetometers [12].

A further wireless localisation system utilises glass-enclosed radio-frequency identification (RFID) tags (LOCalizer™, Hologic Inc., Santa Clara, CA, USA). In this article, we present the findings of our systematic review of the literature regarding LOCalizer™, which shall guide our discussion regarding the merits and issues pertaining to the system, especially in view of the available alternatives. We focused in this review on three aspects: successful deployment of the tags, successful retrieval of the tags and re-excision rates.

## 2. Materials and Methods

### 2.1. Inclusion and Exclusion Criteria

To be included in this systematic review, a study needed to evaluate and report the findings on the use of RFID tags technology to localise non-palpable breast lesions in the abstract.

Only peer-reviewed published articles were included. Retrospective and prospective cohort studies were included. Publications needed to summarise findings when exploring the use of RFID technology to localise non-palpable breast lesions in the abstract. In the full text, the following raw data had to be included: total number of patients undergoing RFID tag localisation, successful placement/localisation of the RFID tags, successful identification/retrieval of the RFID tags and re-excision rates/margin positivity for cancer cases.

Publications comparing RFID tags to other localisation methods were also included. Data regarding other methods were ignored for the purposes of our calculations, except where relevant to the smaller pooled analysis. When available, data regarding re-excision rates were also included. If the publication detailed only margin positivity, this was assumed to indicate re-excision.

### 2.2. Data Sources and Searches

A computer-aided literature search using the EMBASE, Medline and PubMed databases was performed to identify relevant articles for inclusion in the study up to the 4 March 2021 with no lower limit. The following string was used for searching the aforementioned databases: ((breast AND radiofrequency) NOT ablation).

All the titles and abstracts of the studies resulting from the searches were reviewed and articles that were irrelevant were excluded. References of assessed full-text publications were also screened for any relevant publications, as well as previously published reviews. Attempts were made to complete the missing data by asking study authors for the necessary data via electronic mail.

### 2.3. Data Management

The authors extracted and combined data to calculate the overall rates of successful placement and retrieval from data sets of included studies. Some studies included patients who had multiple RFID tags placed for localisation. When no extra data were provided, it was assumed that the number of patients was equal to the number of tags placed.

Mean values were calculated by combining data sets from each included study to give overall rates for successful placement/localisation, retrieval and re-excision. Retrieval rate was calculated using tags successfully removed, whether they were placed accurately or not. Re-excision rate was computed using only cases which had malignancy in their preoperative biopsy or postoperative pathology and had successful tag placement.

A separate analysis was performed on the studies that directly compared the WGL with RFID localisation.

## 3. Results

### 3.1. Literature Search Results and Characteristics of the Included Studies

A total of 814 records were initially identified (239 from PubMed; 353 from EMBASE; 222 from Medline). After removing duplicates, 386 publications were initially assessed for inclusion. All the titles and abstracts were reviewed and articles that were irrelevant, such as studies on radiofrequency ultrasound and radiofrequency spectroscopy were excluded. Conference abstracts, reviews, studies reporting on RFID tags use in localisation of axillary lymph nodes only but not breast lesions were also excluded.

Full texts were then examined for the 10 abstracts, which met the inclusion criteria for our review [10,13,14,15,16,17,18,19,20,21]. One study (Tayeh et al.) [10] was excluded since its results were part of a larger study that is already included in our analysis [19]. Therefore, nine studies were used in the final analysis. Seven cohort studies used to calculate the rates of successful placement, successful retrieval and re-excision in the final pooled analysis [13,14,15,16,17,18,19].

**Figure 1:**
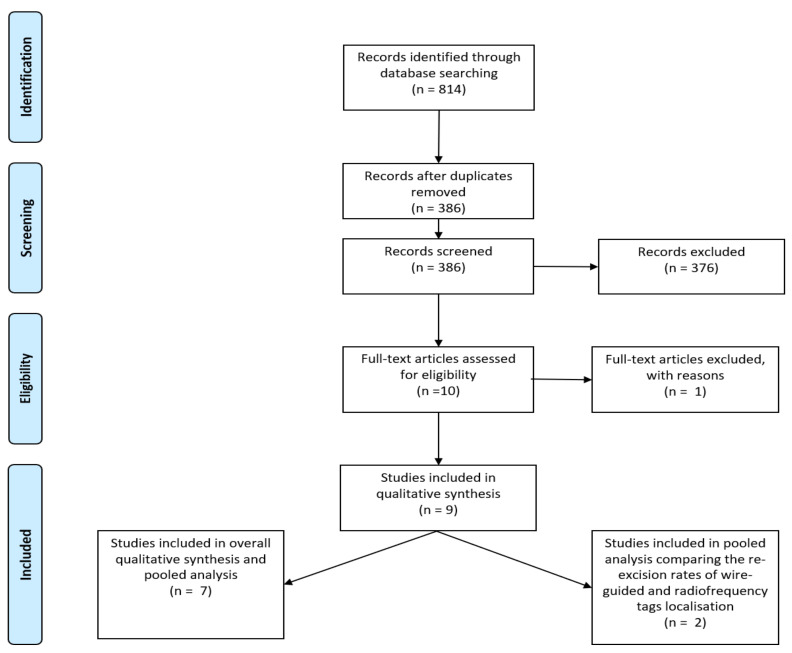
PRISMA flow-diagram illustrating the inclusion and exclusion of studies reviewed for this study.

Two studies compared RFID tags to WGL [20,21]. These were included in a smaller pooled analysis, which selectively investigated re-excision rates in the use of RFID tags in direct comparison to WGL. The results of these two studies were not included in the larger pooled analysis as the cases were from the same authors, institution or study period of two of the seven studies that were included in the larger pooled analysis (Figure 1).

### 3.2. Results of Pooled Analysis

**Figure 2:**
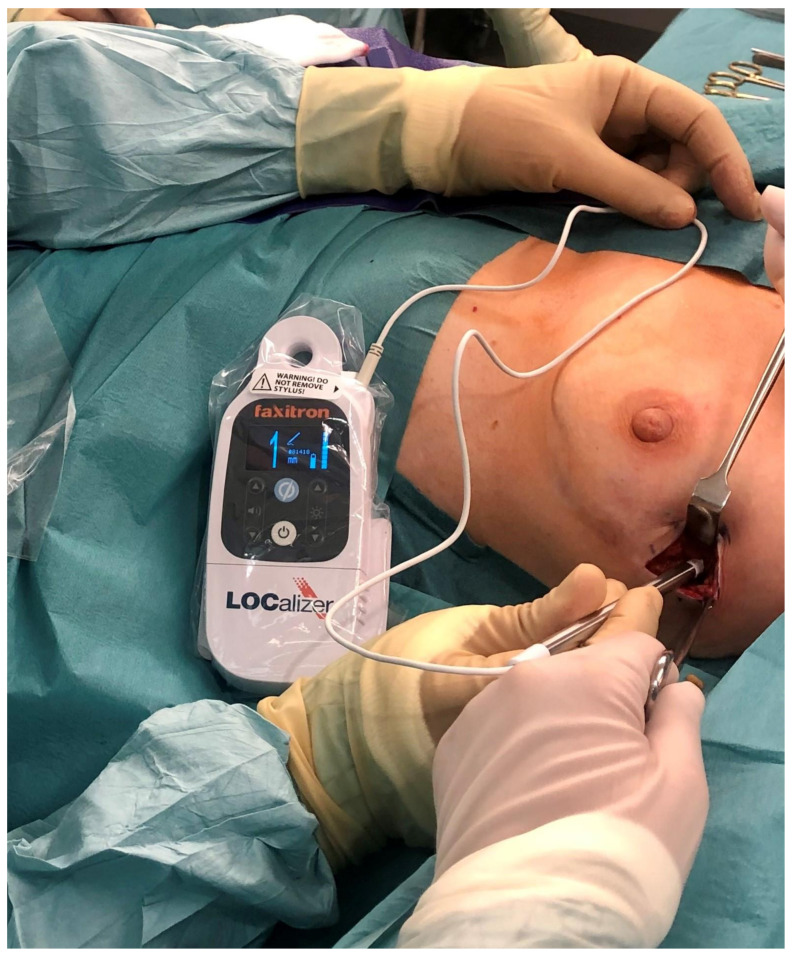
LOCalizer™ handheld reader device being used intra-operatively to locate the RFID tag implanted at the tumour site in the axillary tail of the left breast during surgery. RFID: radio-frequency identification.

Across the nine studies included in the overall pooled analysis, 1344 RFID tags were inserted in 1151 patients. Of these, 1332 were successfully placed and 1343 were successfully retrieved using RFID. This gives a successful deployment rate of 99.1% and a successful retrieval rate of 100%. There were 836 malignant cases with 117 cases requiring re-excision. The re-excision rate was therefore 13.9%. (Table 1) (Figure 2).

Across the two studies directly comparing RFID to wire-guided localisation, 128 RFID tag and 282 wires were inserted to localise malignant lesions. Of these, 20 RFID cases required re-excision compared to 44 wire guided localisation cases. This gives a similar re-excision rate of 15.6 for both techniques. (Chi-squared test, *p* = 0.9954) (Table 2)

Five studies reported no complications [14,15,17,18,19,22]. One study reported a postoperative haematoma which was managed conservatively [13].

Two studies reported on patient, surgeon and radiologist feedback [15,19]. Both studies reported positive feedback. DiNome et al. reported that most patients in their study agreed or strongly agreed (94%) that the procedure went smoothly and agreed or strongly agreed (78%) that the procedure was easier than expected. Radiologists and surgeons suggested that the RFID tag was as fast and reliable as the wire-localised procedure. Surgeons also generally agreed or strongly agreed that the distance gauge was helpful in guiding the surgical dissection [15]. Wazir and colleagues reported patient feedback obtained from seven patients in their study. They reported a mean satisfaction score of 9.9 out of 10 (range = 9–10) using a linear visual analogue scale. In the same study, both radiologists and surgeons rated the LOCalizer™ technique as better compared to wire-guided localization [15].

## 4. Discussion

The limitations of WGL highlighted earlier have given impetus to the development of alternative localisation techniques [23]. Early attempts towards development of an alternative to WGL involved the use of radio-active titanium seeds, which was termed radioactive seed localisation (RSL) [24,25,26]. This method was found to have a favourable learning curve and a low margin positivity rate [27,28]. However, wider acceptance of this modality was curtailed by the regulatory requirements for the handling of radioactive materials. Furthermore, in most jurisdictions, the radio-active markers could be safely retained for only five days, thus requiring coupling of surgical and radiological sessions [29,30].

The limitations of WGL and RSL have inspired the evolution of three wire-free and radiation-free localisation systems which vary on the basis of the underlying principles and technologies [31].

Magseed is based on the detection of a ferromagnetic seed by a proprietary handheld magnetometer called Sentimag which was originally developed for sentinel lymph node and occult breast lesion localisation in the MagSNOLL trial [32,33]. For the purpose of occult breast lesion localisation, a 5 mm seed is implanted using an 18 G introducer. This system was approved by the Food and Drug Administration (FDA) for implantation in 2016 [34,35]. This system compares favourably to WGL in terms of patient acceptability and margin positivity [36,37,38]. MAgnetic MArker LOCalisation (MaMaLoc) (Sirius Medical, Eindhoven, The Netherlands) is another technique based on magnetometry with limited clinical data [39,40,41].

However, magnetometry-centred systems do have some inherent limitations. The markers have been noted to leave significant void signals (>4 cm) in magnetic resonance imaging (MRI) scans, thus limiting their utility in patients who require MRI for their surveillance and diagnosis. Furthermore, the operative field needs to be cleared of ferromagnetic instruments during the use of the magnetometer as they would interfere with the localisation of the seed [12].

SAVI SCOUT is another FDA-approved breast lesion localisation system. This system makes use of proprietary reflectors which reflect electromagnetic signals emitted from and detected by a handheld detector [42,43,44,45]. It has been found to be at least non-inferior to WGL in terms of successful localisation and margin positivity rates. In addition, the device was reported to have minimal MRI void artefacts (<5 mm), in addition to good surgeon, patient and radiologist acceptability [46,47,48].

The LOCalizer™ system is based on ubiquitous RFID technology. It consists of a RFID tag with a unique identification number preloaded in a needle applicator, a surgical probe with 8 mm tip and a portable handheld reader. The tracer chip is encased in glass and is deployed using a 12 G introducer. The surgical probe is the size of a pencil, the reader is portable and displays the distance to the tag in millimetres along with the tag’s ID number (Figure 2). The device is certified for long term implantation. Arguably, using a widespread and well-understood technology would make LOCalizer™ a robust choice.

Our review of the literature identified 1344 devices implanted in 1151 patients. The pooled successful deployment and retrieval rates were 99% and 99.1%, respectively. We found two studies that compared LOCalizer™ with WGL, which found that both modalities had comparable positive margin rates and rates of re-excision. Therefore, it would be reasonable to assert non-inferiority of this modality compared to the current gold standard of WGL in terms of successful localisation and margin positivity rates with the added advantage of flexible scheduling. However, none of the primary studies included in our review was a randomised controlled trial. Moreover, every tag has a unique identification number which is particularly valuable when multiple markers are deployed for localisation.

**Figure 3:**
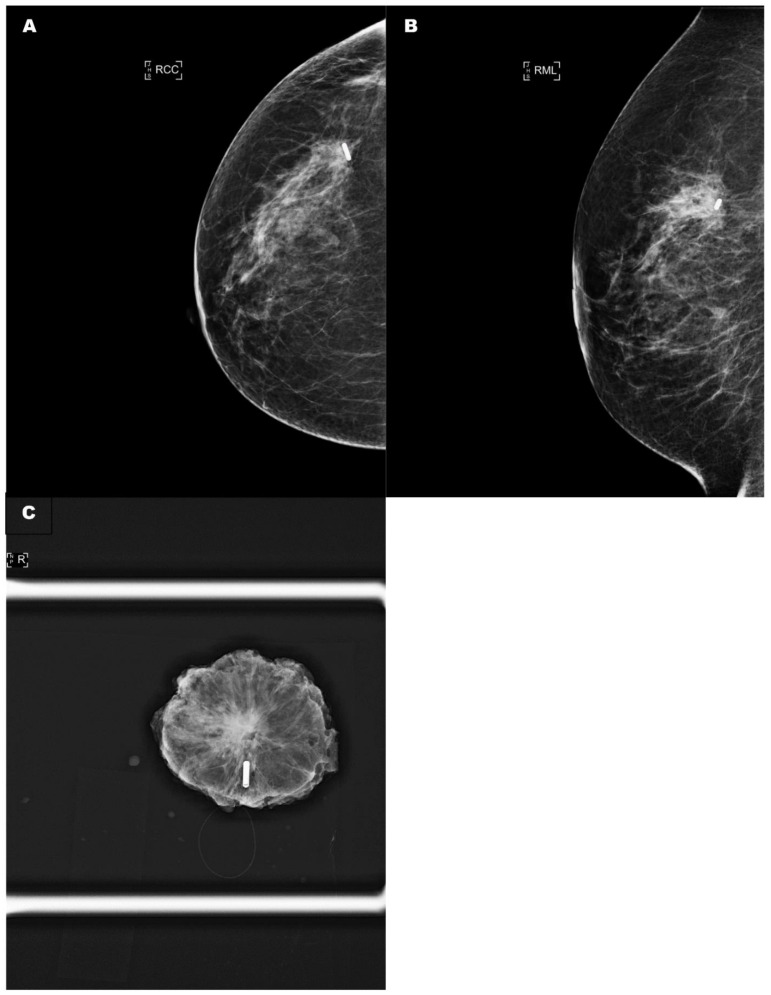
(**A**) A control craniocaudal mammography film showing the radio-frequency tag within the tumour. (**B**) A control oblique mammography film showing the radio-frequency tag within the tumour. (**C**) A specimen radiograph demonstrating that the radio-frequency tag had migrated by 1 cm along the needle introducer track. RCC: right craniocaudal; RML: right medialateral.

However, the LOCalizer™ system has certain limitations worth highlighting. First, the tags cause void artefacts in MRI scans, albeit smaller than Magseed, which can have implications for post-deployment imaging in certain patients especially in the context of neoadjuvant systemic therapy (NST) [49]. Second, the detection range of the system is limited to 6 cm only, potentially causing issues in larger breasts and deeper lesions [50]. Third, the current LOCalizer™ introducer needle is wider than that of Magseed and Savi Scout. Therefore, deployment can be difficult, particularly in dense breast tissue [14,50]. Furthermore, the wide bore introducer needle (12 G, or 2.77 mm) often requires a skin incision for insertion and can create a wide track along which the glass-encased tag could migrate (Figure 3). Although studies have not reported significant migration before surgery, migration has been reported to occur during excision of the surgical specimen. Dauphine et al. observed migration in 3 (15%) of 20 cases, as the lesion was being retracted with fingers to make the final cut along the deep surface of the specimen [14]. Lamb et al. have also implicated the wide gauge introducer needle in the loss of titanium clip markers during tag deployment in some cases [16]. Finally, patients with cardiac pacemakers and defibrillators should be excluded as a precautionary measure since radiofrequency signals may interfere with the function of these devices [14].

Therefore, the LOCalizer™ technology can be further optimised in its design to improve clinical performance. We previously suggested that the bore of the introducer needle should be reduced to 16 gauge in order to facilitate deployment within dense tissue and reduce the probability of tag migration along the introducer needle track [19,21]. We also recommend that removal of the glass casing of the RFID tag should be considered since it contributes to its migratory potential and MRI void artefacts [14]. Furthermore, retention of glass fragments can occur in the case of tag fracture.

In addition to this review, we previously reported the results of a pooled analysis of all published studies that investigated the use of SAVI SCOUT [11] and Magseed [12] and reported an overall successful localisation rate of 99.64% and 99.86%, respectively. We found no significant difference in re-excision rates when comparing Magseed with WGL [10,12]. However, our pooled analysis of SAVI SCOUT performance reported that the use of the device was associated with a significant reduction in the re-excision rates compared with WGL in subgroup analysis [9,11,46]. However, this requires further validation in adequately powered prospective studies.

The strength of our review stems from the fact that it represents the first pooled analysis of all published studies reporting experience with the use of RFID technology for the localisation of more than 1000 non-palpable breast lesions thus providing an overview of its clinical performance. Our findings confirm the safety of this wire-free approach and will be helpful for the design of future prospective studies. We have also proposed certain technical modifications of the deployment system and the tag in order to optimise their clinical performance.

Our study has several limitations. Although we included nine studies in our systematic review, most of the data arose from a single retrospective analysis with paucity of data from prospective trials [16]. None of the studies was a prospective randomized trial. The number of patients in most of the primary studies was relatively low (<100). Only two studies included more than 100 patients [16,17]. Only one small study compared RFID with other wireless, radiation free technologies [20].

Furthermore, most of the cases included in our analysis had the radio-frequency tag deployed on the day of surgery, thus making it difficult to draw conclusions regarding the clinical performance of the technology with regards to the intended decoupling of surgical and radiological scheduling. This will also compromise the evaluation of tag migration over time which is an important consideration.

Moreover, the RFID tags were deployed using mammographic guidance to localise lesions visible on ultrasonography in a large number of cases [16], thus prolonging the duration of the localisation procedure and increasing radiation exposure.

The retrospective and heterogeneous nature of most primary studies in addition to lack of methodology standardisation precluded a meaningful analysis of important secondary outcomes such as the depth of the lesion from the skin surface, the weight of the surgical specimen, duration of procedure, the size of MRI void signals and aesthetic considerations.

Finally, none of the studies included have evaluated the cost-effectiveness of using this technology.

## 5. Conclusions

Our systematic review includes 1151 patients and 1344 RFID tags. The pooled deployment rate was 99.1% and retrieval rate was 100%. Re-excision rate was 13.9%. In studies comparing RFID with WGL, the re-excision rate was comparable.

In summary, our review confirms that the RFID-based LOCalizer™ system is a valid safe alternative to WGL in terms of successful localisation and margin positivity with the added advantage of decoupling surgery and radiology scheduling. However, the system requires technical refinements to optimise its clinical performance, and its impact on aesthetic outcome and healthcare economics should be evaluated in future prospective research.

## Figures and Tables

**Table 1:** Details of studies included in pooled analysis. CI: confidence intervals; RFID: radiofrequency identification.

References	Author	Country	Year of Publication	Number of Patients	Number of Patients with Breast Cancer	Number of RFID Tags Deployed	Number of RFID Tags Successfully Deployed (Successful Deployment Rate)	Number of RFID Tags Successfully Retrieved Surgically (Retrieval Rate)	Number of Patients Requiring Re-Excision/Number of Patients with Breast Cancer (Re-Excision Rate)
[9]	Cullinane	Ireland	2020	69	63	69	69 (100%)	69 (100%)	12/63 (19%)
[10]	Dauphine	USA	2015	20	15	20	20 (100%)	20 (100%)	4/15 (26.7%)
[11]	DiNome	USA	2019.	50	33	50	50 (100%)	50 (100%)	2/33 (6.1%)
[12]	Lamb	USA	2020	848	568	1013	1004 (99.1%)	1012 (100%) *	86/568 (15.1%)
[13]	Lowes	UK	2020	150	150	177	174 (98%)	177/177 (100%)	13/150 (8.7%)
[14]	Malter	Germany	2019	4	0	4	4 (100%)	4 (100%)	All benign
[15]	Wazir	UK	2020	10	7	11	11 (100%)	11 (100%)	0/7 patients (0%)
Total				1151	836	1344	1332/1344(99.11%) 95% CI: 98.45–99.54	1343/1343 (100%) 95% CI: 99.73–100.00	117/836 (13.9%) 95% CI: 11.71–16.53

* One patient with one tag did not have surgery due to metastatic disease (information from the author’s personal communication).

**Table 2:** Details of studies included in pooled analysis comparing re-excision rate between radio-frequency identification (RFID) tag localisation and wire-guided localisation (WGL). (Chi-squared test, *p* = 0.9954).

References	Author	Year of Publication	Number of Patients with Breast Cancer Undergoing RFID Tag Localisation	Number of Patients with Breast Cancer Undergoing RFID Tag Localisation Requiring Re-Excision (Re-Excision Rate)	Number of Patients with Breast Cancer Undergoing WGL	Number of Patients Undergoing WGL Requiring Re-Excision (Re-Excision Rate)
[16]	Lee	2020	33	2 (6.1%)	50	5 (10%)
[17]	McGugin	2019	95	18 (18.9%)	232	39 (16.8)
Total			128	20 (15.6%) 95% CI: 11.57–20.37	282	44 (15.6%) 95% CI: 9.81–23.10

## Data Availability

Publicly available datasets were analyzed in this study.
